# Study of the Lipophilicity of Tetracyclic Anticancer Azaphenothiazines

**DOI:** 10.3390/biom15081194

**Published:** 2025-08-19

**Authors:** Małgorzata Jeleń, Beata Morak-Młodawska, Małgorzata Dołowy, Adam Konefał

**Affiliations:** 1 Department of Organic Chemistry, Faculty of Pharmaceutical Sciences in Sosnowiec, Medical University of Silesia in Katowice, Jagiellońska Street 4, 41-200 Sosnowiec, Poland; bmlodawska@sum.edu.pl; 2 Department of Analytical Chemistry, Faculty of Pharmaceutical Sciences in Sosnowiec, Medical University of Silesia in Katowice, Jagiellońska 4, 41-200 Sosnowiec, Poland; mdolowy@sum.edu.pl; 3 Institute of Physics, University of Silesia in Katowice, 40-007 Katowice, Poland; adam.konefal@us.edu.pl

**Keywords:** anticancer quinobenzothiazine, RP-TLC, lipophilicity, chlorpromazine, phenotiazines

## Abstract

Although chlorpromazine is primarily used in psychiatry, it has been shown since its introduction to influence the course of neoplastic diseases. According to the strategy of drug repurposing, chlorpromazine has been successfully tested for its potential antitumor effects on multiple cancer cell lines. This effect is consistent with the overlap of molecular pathways observed for years between schizophrenia and cancer. The main objective of this work was to evaluate the lipophilicity of 17 previously synthesized tetracyclic chlorpromazine analogues exhibiting diverse anticancer and antimicrobial activity using thin-layer chromatography and computational methods. For a compound to become an effective drug, it must have a favorable ADMET profile, which determines its pharmacokinetic properties as a drug candidate. Lipophilicity is one of the key parameters widely employed in designing new bioactive compounds as potential therapeutic agents. In this article, chromatographic plates precoated with silica gel 60 RP-18F_254_ and a mixture of acetone and TRIS buffer were used as the mobile phase. The chromatographic parameter of lipophilicity (R_M0_) of the investigated compounds determined by means of the Soczewinski–Wachtmeister formula was useful to obtain the values of the experimental lipophilicity parameter expressed as logP_TLC_. The results of logP_TLC_ were compared with theoretical values of logP obtained using different algorithms (iLOGP, XLOGP3, WLOGP, MLOGP, SILCOS-IT, and ClogP). Furthermore, the online platforms, such as SwissADME and pkCSM, allowed the determination of the remaining ADME parameters of the quinoline derivatives of chlorpromazine. The study of lipophilicity and ADME factors enabled confirmation that the tested compounds demonstrated favorable properties. Therefore, they can be considered as promising starting structures for further studies.

## 1. Introduction

Phenothiazines (PTHs) are heterocyclic compounds based on sulfur and nitrogen. Due to their high biological activity, the phenothiazine ring system is one of the leading structures in the pharmaceutical industry particularly in the development of new drugs [1,2,3]. A comprehensive review of the literature shows that phenothiazine derivatives have wide application in both psychiatric states (e.g., antipsychotic, neuroleptic) and non-psychiatric states, such as antiviral or anti-proliferative effects [1,2,3]. Chlorpromazine belongs to the aliphatic group of phenothiazines. This compound was introduced to psychiatric practice in 1952 and has influenced advances in the treatment of mental illness. Over the past few decades, much attention has been paid to the synthesis of phenothiazine derivatives and their study for a variety of pharmacological activities, including antibiotics, painkillers, sedatives, and antivirals [1,2,3]. In recent years, in vitro studies have confirmed also its usefulness as a promising antitumor agent showing activity against various cancers, such as glioma [4]. Other studies have suggested that work has pointed to the use of this drug to combat psychotic episodes in patients diagnosed with COVID-19, alone or in combination with hydroxychloroquine [5,6,7,8]. The compounds containing chlorpromazine have also been shown to have potentially anticancer properties against the cancer cells of colon, breast, lung, brain, as well as leukemia and lymphoma [5,6,7,8].

These observations highlight the need to develop new phenothiazine derivatives, including chlorpromazine analogs, as effective drugs against these diseases. For a new molecule, including phenothiazine derivatives, to become an effective drug, it must not only exhibit biological activity, but also be safe for the potential patient and have a favorable ADME (absorption, distribution, metabolism, and excretion) profile which determines the pharmacokinetic properties of individual bioactive compounds as drug candidates. The ADME parameters need to be predicted early, during the first stages of drug development. This can help to reduce the number of compounds with undesirable ADME properties studied in later phases of drug development, i.e., in clinical trials. Among the various physicochemical properties, lipophilicity is one of those that can affect the ADME profile of active molecules. Lipophilicity is a key parameter in drug design, and thus in the pharmaceutical industry, because it determines the permeability of the compound through the membrane in the biological system and is helpful in optimizing the structure of new drug molecules [9,10,11,12,13]. Lipophilicity of organic compounds is expressed as log P, the base-10 logarithm of the partition coefficient (P), which is defined as the ratio of the compound’s concentration in two immiscible solvents: a non-polar organic phase and a polar aqueous phase at equilibrium [9]. Current methods of determining the lipophilicity parameter include, in addition to the traditional flask shaking technique, various chromatographic systems for thin-layer chromatography and high-performance liquid chromatography (HPLC—high-performance liquid chromatography) [9,14,15]. In this method, lipophilicity chromatographic parameters (R_M0_ and logk_w_) are obtained from the retention factor R_M_ or k and extrapolated to the zero content of the organic modifier in the applied mobile phase. Due to its ease of use and the possibility to analyze several compounds simultaneously on the same plate, TLC (thin-layer chromatography) is a method often used in the study of the lipophilicity of different groups of newly synthesized compounds as potential drug candidates. Numerous applications of thin-layer chromatography in the evaluation of the lipophilicity of pharmaceuticals including potential candidates for new drugs are reported in the literature [16,17,18,19,20,21,22]. In addition to experimental methods, a wide range of computational tools for predicting ADME parameters, including lipophilicity, have been developed in recent decades, such as SwissADME, pkCSM, Molinspiration, and others [23,24,25,26,27,28,29]. Existing calculation methods for log P are classified into four groups:

- atom-based methods;
- fragment-based methods;
- topology-based methods;
- structural-property-based methods.

It is known that the logP computational algorithms from different families have their respective advantages and disadvantages. Some calculation methods are more or less suited for specific heterogeneous compounds. The poor predictive power of software packages might be explained by insufficient coverage of the chemical surface by measured compounds. The calculation algorithms are as good as the data that they are based on. Generally, the additional corrections introduced to them allows one to achieve better prediction accuracy for the determination of the theoretical parameter of lipophilicity. Correction factors are introduced to rectify the calculated logP when some special substructures occur in the molecule. As it was well described by Mannhold et coworkers [30], there are structural and interaction factors. Structural factors concern the chain bonds, ring bonds, and branch bonds. Interaction factors consider aliphatic proximity, electronic effects through π-bonds as well as special ortho effects [30]. The first type of algorithms, i.e., atom-based methods and atom additive methods such as AlogP, are suitable for small molecules without complex structures. These methods cut molecules down to single atoms and do not apply correction rules. An advantage of atom-based methods is that ambiguities are avoided; a shortcoming is the failure to deal with long-range interaction [30]. Therefore, to overcome these shortcomings, the adjusted atom-based or hybrid methods are implemented like XlogP or Silicos-IT logP, respectively. Currently, a new version of the XlogP3 algorithm adopts an optimized classification scheme of 87 atom types as well as two correction factors accounting for internal H-bonds and amino acids. Fragment-based methods divide molecules into fragments and apply correction factors to account for intramolecular interactions (e.g., ClogP) for log P calculation are similarly based on summing up of the hydrophobicity contribution of each fragment in a molecule, i.e., fragment constant (hydrophobicity contribution of each fragment) which are determined by the experimental value of log P. The above-mentioned Clog P is the most frequently used log P calculation tool. Recent versions include the basic fragmental values which were derived from the measured log P data of simple-molecule complex hydrocarbons, whose measured values were not the sum of the fragment values; the differences were defined in terms of correction factors. Thanks to the additional correction factors taking into account additional interactions, such as hydrophilicity shield effect and hydrogen bonding, these methods are better predictors for large molecules. Next, the third family is topology- or graph-based methods (e.g., Mlog P) which use topological descriptors generated by means of 2D structures. Their main advantage is speed. The last group is structural-property-based methods. These methods use a physical–chemical perspective and 3D structure to calculate log P values. Among the computational methods, DFT-based implicit solvent models provide a physically grounded approach by estimating solvation free energies in water and 1-octanol to compute log P. Owing to systematic error cancelation between solvents, they offer reasonable accuracy (typically with mean absolute errors of around 0.6 log units) and are computationally more efficient than explicit solvent simulations. Although generally outperformed by empirical fragment-based methods for well-represented neutral compounds, DFT-based models are advantageous when dealing with novel or structurally diverse molecules outside standard training sets [31].

Prediction of lipophilicity parameters and other ADME descriptors in silico allows for early reduction of new drugs with undesirable ADME properties. In several papers, experimental values of lipophilicity parameters were compared with theoretical values of the partition coefficient obtained using software and web servers to predict ADME parameters [17,20,21,22,29]. Theoretical values of the lipophilicity descriptors of the studied compounds may be helpful. However, predictions will not be enough without the experimental evaluation of drug candidates during the drug discovery and development process.

**Figure 1:**
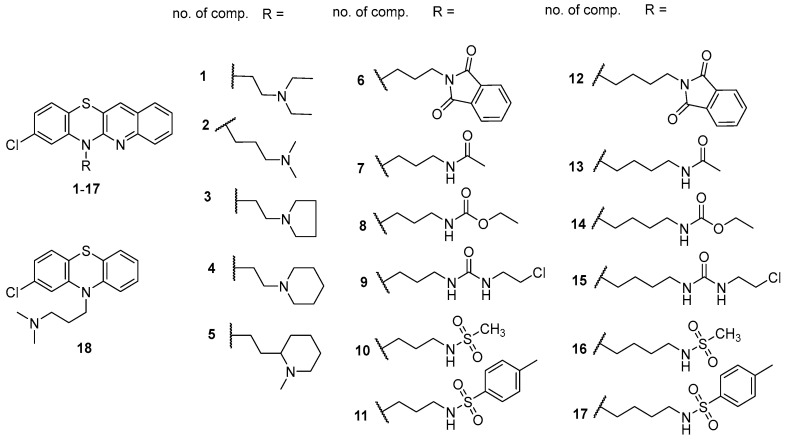
Chemical structure of tested compounds **1**–**17** and chlorpromazine **18**.

Thus, our work aimed to determine the lipophilic parameters of previously synthesized chlorpromazine derivatives (**1**-**17**)—Figure 1—both experimentally using thin-layer reverse phase chromatography (RP-TLC) and computationally, employing various software tools. In addition, other key ADMET parameters that describe the pharmacokinetic behavior of drugs were determined. Chlorpromazine (**18**) (known for years as a drug) was also used in the studies as a reference compound to compare the tested parameters of the new substances. The results of our previous work confirmed the antibacterial and pro-apoptotic activity of these novel compounds in relation to the following cancer cell lines: A549, MiaPaCa-2, and HCT-116 [32].

## 2. Results and Discussion

This study presents an analysis of the lipophilic properties and other ADME parameters obtained in silico for quinoline analogues of chlorpromazine that were previously synthesized and exhibit diverse anticancer and antimicrobial activity. The new derivatives were obtained via multi-step reactions based on 6*H*-8-chloroquinobenzothiazine [32]. This tetracyclic phenothiazine was obtained via the reaction of 2-amino-4-chlorobenzenethiol and 3-bromo-2-chloroquinoline. The tested 8-chloroquinobenzothiazines exhibited promising activity against A549 cells without affecting HaCaT cells. Compounds **2**, **9**, **15**, and **17** (Figure 1) showed the most promising cytotoxicity against A549 cells and a higher selectivity index (SI = 7.6–10.7) than the reference compound, doxorubicin (SI = 0.14–0.15). Compounds **2** and **16** showed the highest selectivity index (143), with IC_50_ values of 1.6 µM and 0.7 µM, respectively, against HCT-116 cells, while showing no cytotoxic effects on HaCaT cells. Compound **5**, on the other hand, showed high cytotoxicity against HCT-116 cells (7.7 µM) (Table S1). Studies on the mechanisms of cytotoxic action of the discussed substances confirmed their proapoptotic activity, especially in terms of inducing late apoptosis or necrosis in cancer cell lines A549, MiaPaCa-2, and HCT-116. The activity of new chlorpromazine analogues against standard Gram-positive bacteria (various strains of *S. aureus* and *S. epidermidis*) and Gram-negative bacteria (*E. coli* and *P. aeruginosa*) was also tested. 8-Chloroquinobenzothiazines **1**, **2**, **3**, **9**, and **15** showed moderate antibacterial activity, mainly against standard strains of staphylococci. The most significant activity against standard strains was observed for compound **15** (MIC = 2–8 µg/mL) [32].

Studies to determine and analyze the lipophilicity parameters of the new substances began by determining the lipophilicity parameters (log P_calc_) using selected computational programs. The values obtained computationally are presented in Table 1.

Analyzing the obtained results, one can observe large differences in the values of the lipophilicity parameters calculated with different programs, i.e., log P_calc_. Differences in the values obtained for individual substances reach even more than three units. For example, for 8-chloroquinobenzothiazine **17**, the iLOGP program provided a value of 3.67, while the WLOGP and Clog P programs yielded 7.26 and 7.81, respectively. In the case of substance **14**, the MLOGP program produced a value of 3.04, and the Clog P program yielded 6.76. For most of the tested substances, the lowest values for log P were obtained using the iLOGP program. The exceptions were 8-chloroquinobenzothiazines **14** and **16** for which the lowest values of the log P_calcd_ parameter were calculated using the MLOGP program. The highest values of this parameter reaching up to 7.90 for compound no **12** were obtained from the Clog P program. The calculated log P values were not sufficiently precise in terms of possible contributions from conformation, folding, hydration, ion pair formation, and intra- and intermolecular hydrogen bond formation. The low predictive power of the programs can be attributed to the nonplanarity of the four-ring quinobenzothiazine system as well as the boat conformation of the central thiazine ring (such conformation was determined via X-ray analysis of the selected compounds and may additionally result from an unpredictable conformation of long substituents at the thiazine nitrogen atom. These results show that, although computational techniques are a cheap, fast way to estimate lipophilicity parameters of new drug candidates, especially in early stages of research, experimental methods still cannot be omitted.

In connection with this, in the next stage of the study, the parameters of lipophilicity of the tested compounds were determined experimentally. The experimental lipophilicity of substances **1**–**18** was tested using the RP-TLC method. This technique was used to determine the retardation coefficient R_f_ for each compound in eight mobile phases consisting of acetone and Tris buffer in different volume ratios (50–85% acetone). Next, the R_f_ values were converted to the chromatographic parameter R_M,_ and then the obtained values were extrapolated to 0% acetone content in the mobile phase obtaining the lipophilicity parameter R_M0_ (Table S2). In addition to this, due to a relationship observed between R_M0_ and the slope of these linear plots (b), the lipophilicity parameter C_0_ was also determined (Table S2).

The parameter R_M0_ can be correlated to an experimental log P lipophilicity parameter of chemical molecules (log P_TLC_) by applying a calibration curve. The calibration curve was obtained by determining the R_f_ value for standard substances with a known log P_lit_ value using the RP-TLC method under conditions analogous to those of the tested substances. After calculating the R_M_ and R_M0_ values, the dependence of the log P_lit_ parameter on the experimentally obtained R_M0_ values for standards was determined (Table 2).

A relationship characterized by a high correlation coefficient was obtained. Using the standard curve equation:

log P_TLC_ = 1.2862R_M0_+ 0.2061 (r = 0.9999; s = 0.003; F = 1864523.15; *p* = 0.001)

log P_TLC_ parameters for the tested compounds **1**–**18** were calculated. The log P_TLC_ values of the tested compounds obtained in this way are presented in Table 3. The experimentally determined log P_TLC_ values for the tested quinoline analogues of chlorpromazine ranged from 3.70 to 6.22. The highest lipophilicity was determined for derivative **17** with a *p*-toluenesulfonamidobutyl substituent at the thiazine nitrogen atom and the lowest for derivative **7** with an acetylaminopropyl substituent. The values of the log P_TLC_ parameter for the tested substances **1**–**5**, containing dialkylaminoalkyl substituents, were similar and were in the range of 3.77 to 4.20. Replacing the pyrrolidine ring (compound **3**) with a piperazine ring (compound **4**) did not affect this parameter’s value. A greater change can be observed by changing the substituent to 1-methyl-2-piperidine (compound **5**). Among derivatives **6**–**11** containing differently substituted propyl fragments and for derivatives **12**–**17** with four-carbon linkers, the highest values of the log P_TLC_ parameter were obtained for derivatives **11** and **17** containing a *p*-toluenesulfonamide fragment. In addition, compounds with the butylene chain (**6**–**11**) were more lipophilic than compounds with the propylene linker (**12**–**17**). In our previous studies on the lipophilicity of a large group of 6,9-disubstituted quinobenzothiazines (with H, Cl, and SCH_3_ at position 9 of the quinobenzothiazine ring) we found the same pattern [38]. The lipophilicity of derivative **2** with a dimethylaminopropyl substituent was slightly higher than the lipophilicity of chlorpromazine **18** used as a reference substance. The introduction of the quinoline system in place of one of the benzene rings of the thiazine system increased the value of the lipophilicity parameter by nearly 0.5.

**Figure 2:**
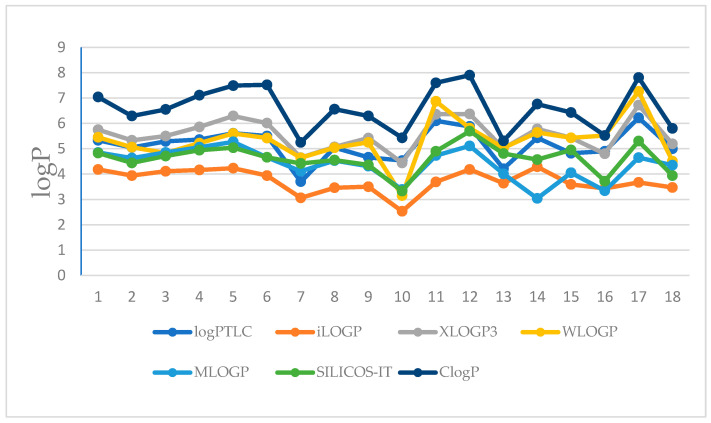
Comparison of theoretical parameters of lipophilicity (log P_calc_) and experimental values (log P_TLC_) of tested 8-chloroquinobenzothiazines **1**–**17** and chlorpromazine **18**.

Figure 2 shows a comparison of all theoretical lipophilicity parameters (log P_calc_) and experimental (log P_TLC_) of the examined 8-chloroquinobenzothiazines **1**–**17** and chlorpromazine **18**.

**Figure 3:**
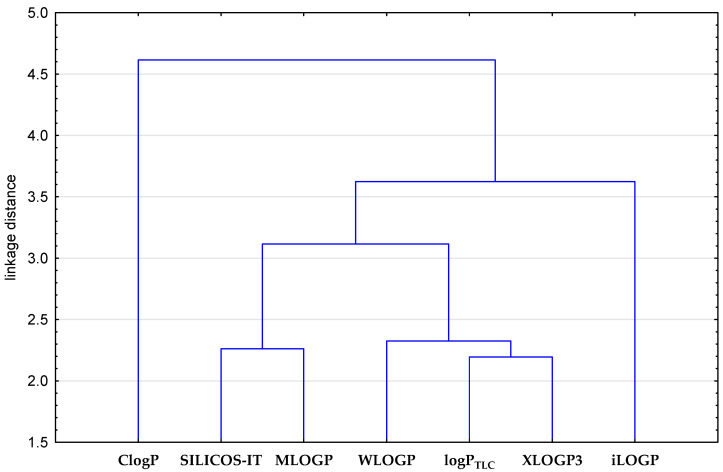
Dendrogram of similarity of lipophilicity parameters of examined compounds **1**–**17** (8-chloroquinobenzothiazines) and chloropromazine **18**.

As can be seen in the graph shown in Figure 2, the closest log P values (overlapping curves) were obtained using computational algorithms such as XLOGP3, WLOGP, MLOGP, and SILCOS-IT, as well as the RP-TLC method as log P_TLC_ for compounds **1**–**7**. This fact indicates that the four different algorithms used to calculate log P are suitable for the rapid estimation of the lipophilicity of the first group of tested compounds designated as **1**–**7**. The analysis of Figure 3 confirms the previous observations regarding the different power of prediction of computational algorithms used to calculate the theoretical value of the partition coefficient of the studied compounds in relation to the RP-TLC method. Therefore, in order to compare both, in the further stage of the analysis, the experimental log P_TLC_ values of all tested compounds were compared with the calculated values (log P_calc_) using a statistical tool, namely cluster analysis. Interpretation of dendrogram of all lipophilicity parameters of examined compounds **1**–**17** (8-chloroquinobenzothiazines) and chloropromazine **18** presented in Figure 3 shows that the biggest similarities in the case of all compounds indicate the theoretical log P values obtained by means of SILICOS-IT and Mlog P algorithms. These two partition coefficients form one cluster with the smallest Euclidean distance. The observed similarity of experimental values of the partition coefficient as log P_TLC_ with Xlog P3 values confirms that this model of calculation based on the atom-based method including corrective factors can be a good tool to obtain reliable results of lipophilicity parameters for all the compounds studied.

**Figure 4:**
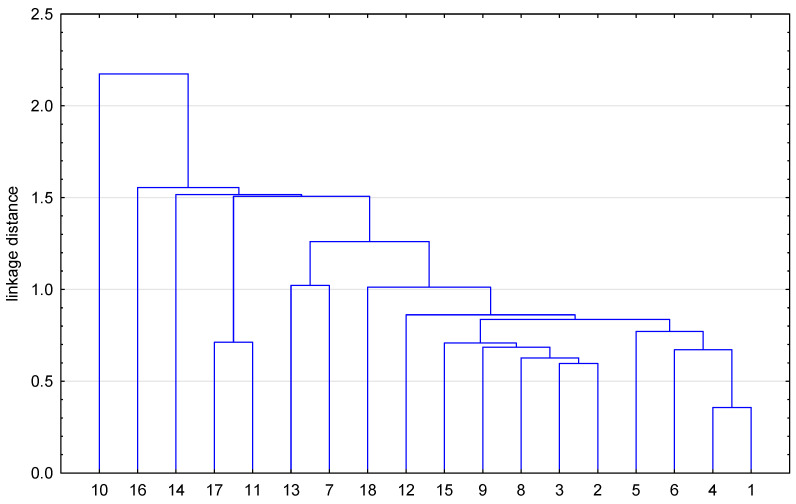
Dendrogram of similarity of tested compounds **1**–**17** (8-chloroquinobenzothiazines) and chloropromazine **18** based on their lipophilicity.

Figure 4 shows the dendrogram of similarity of the tested compounds (8-chloroquinobenzothiazines) **1**–**17** and chloropromazine **18** based on both lipophilicity parameters, i.e., theoretical (Clog P, XLOGP3, WLOGP, MLOGP, SILICOS-IT, iLOGP) and chromatographic one (log P_TLC_).

Analysis of the dendrogram shown in Figure 4 indicates that cluster analysis allowed one to group all tested compounds **1**–**18** into several smaller and then one large cluster taking into account the lipophilicity properties as the key parameter in describing the ADME profile of the studied compounds. The highest similarity given lipophilic properties indicates compound **4** and **1** (the smallest Euclidean distance) with diethylamine and piperidine substituents, respectively, and derivatives **2** and **3** with dimethylamine and pyrrolidine substituents. Substance **5**, also containing a dialkylaminoalkyl substituent, shows less similarity to compounds **1**–**4**. This may be due to the different position of the nitrogen atom in the piperidine ring than in substance **4**. A common cluster, although isolated from the rest, is formed by substances **11** and **17** containing a *p*-toluenesulfonic fragment in the substituent at the thiazine nitrogen atom, and substances **7** and **13** with acetylaminoalkyl substituents. On the other hand, the smallest similarity to other compounds (the greatest distance) is observed at the dendrogram in the case of compound **10**.

**Figure 5:**
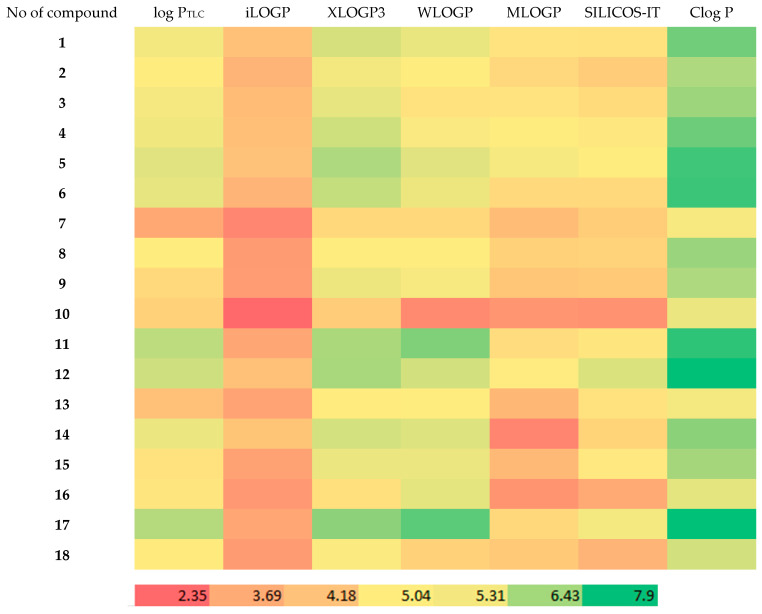
Heat map showing the relationship between theoretical and chromatographic lipophilicity parameters of the tested compounds.

Next, to illustrate the relationship between the theoretical partition coefficients and the chromatographic (i.e., experimental) lipophilicity parameter (log P_TLC_), a heat map was generated (Figure 5).

As can be seen in Figure 5, the Clog P parameter was found to be the closest to the experimental value. The MLOGP parameters were found to deviate the most from the log P_TLC_ value. A significant similarity can also be observed in the values obtained with the XLOGP program. These log P values were calculated by means of atom-based and fragment contribution methods [27]. Correlating experimentally determined and calculated log P values remains crucial for understanding and predicting lipophilicity. As demonstrated in recent systematic studies of a series of fluorinated compounds, computational methods—from fragment-based models to quantum mechanical approaches—can reflect general trends in lipophilicity, although discrepancies often arise for specific structural motifs. These differences underscore the importance of careful model selection and validation against experimental data when investigating the effects of subtle chemical modifications on log P [39].

In order for a newly synthesized substance to be considered as a potential drug candidate, it must exhibit specific pharmacokinetic, pharmacological, and toxicological properties. To assess the suitability of a given substance as a drug candidate, it is necessary first to assess its similarity to the drug and determine its properties in terms of absorption, distribution, metabolism, and excretion (ADME). A preliminary assessment of the similarity of a molecule to the drug is performed early in the research process to accelerate the discovery and development of new drugs. There are several methods for assessing the drug similarity of a tested substance. These include the rules proposed by Lipinski, Ghose, Veber, Egan, and Muegge. Each of these rules aims to determine whether a chemical compound with a specific pharmacological or biological activity has properties that make it an active orally administered drug. The criteria on which these rules are based refer to the properties of the substance that are relevant to pharmacokinetic processes (absorption, distribution, metabolism, and excretion). In order to confirm the bioavailability of the tested compounds **1**–**18**, their compliance with the rules of Lipinski, Ghose, Veber, and Egan was checked using the SwissADME platform. The bioavailability parameters that allow us to determine the drug-likeness of the tested substances are presented in Table 4.

The molar mass of the tested compounds **1**–**17** is in the range of 369.91 (compound **2**) to 510.07 (compound **17**), the value of the lipophilicity parameter MLOGP is in the range of 3.04 (for substance **14**) to 5.27 (for substance **5**), the number of n-HBA hydrogen bond acceptors for all substances does not exceed 10, and for each of the tested compounds **1**–**17**, the number of hydrogen bond donors is below 5. Lipinski’s rule assumes that a substance with good bioavailability should have a molecular weight < 500, no more than five hydrogen bond donors, no more than ten hydrogen bond acceptors, and a lipophilicity parameter value MLOGP < 5. A substance is considered to meet this rule if it exhibits three of these parameters. The results of fitting the tested substances to the Lipinski rule are presented in Table 5. Of the substances tested, only compound **17** does not comply with this rule. The table also shows which parameters fall outside the range given for the Lipinski rule. Because the Lipinski Rule of Five (Ro5) remains a common benchmark for assessing oral drug similarity, we decided to address it in this study, although its limitations are increasingly recognized. As the recent literature has shown, Ro5 should be viewed as a flexible guideline rather than a strict rule, especially given its limited applicability to certain classes of compounds [40]. More advanced metrics, such as ligand lipophilic efficiency (LLE), molecular flexibility, and three-dimensionality, now offer a more nuanced understanding of drug similarity in modern lead optimization. According to Ghose’s rule, a drug-like substance should have a molecular weight in the range of 160–480, a molar refractive index in the range of 40–130, a WLOGP of 0.4 to 5.6, and an atom count of 20 to 70. As shown in Table 5, six of the seventeen substances tested do not meet these criteria (**5**, **6**, **11**, **12**, **14**, and **17**). However, all the 8-chloroquinobenzothiazines tested meet Veber’s rule, which assumes that a substance that is a good drug candidate should have at most 10 rotatable bonds and a TPSA of at most 140 Å. Egan’s rule based on the parameters TPSA and WLOGP (WLOG ≤ 5.88, TPSA ≤ 131.6 Å^2^) is not fulfilled only by substances **11** and **17** with *p*-toluenesulfonamidoalkyl substituents. However Muegge’s rule is fulfilled only by three of the tested substances, 8-chloroquinobenzothiazines **7**, **10**, and **16**. This rule is based on the highest number of parameters, and the tested substances do not meet it due to the value of the lipophilicity parameter XLOGP3 being higher than 5 [41]. In terms of similarity to chlorpromazine used as a reference, which can be assessed based on the rules of Lipinski, Ghose, Veber, Egan, and Muegge, the tested derivatives containing dialkylaminoalkyl substituents **1**–**4** and derivatives containing three-carbon chains in the substituent at the thiazine nitrogen atom **7**–**10** may exhibit similar properties (Table 5).

In order for a newly obtained chemical molecule to be considered as a potential drug, it must demonstrate a favorable ADME profile, good bioavailability, and no side effects. Predictive methods are inexpensive and convenient for the initial assessment of these parameters at the first stage of the evaluation of new substances in terms of their suitability as potential therapeutic substances [42].

To initially assess the ADME profile of the synthesized compounds, pkCSM was used, a program that is often used in the initial evaluation of new drug candidates [43]. Using this program, the parameters responsible for absorption (water solubility, Caco-2 permeability, intestinal absorption, skin permeability), distribution (VDss, unbound fraction, BBB permeability, and CNS permeability), excretion, and toxicity (total clearance, max. tolerated dose, oral rat acute toxicity, oral rat chronic toxicity, T. Pyriformis toxicity, and minnow toxicity) were calculated for the discussed substances.

The parameter concerning water solubility obtained from the pkCSM program is given as logS (S—solubility expressed in mol/L). All tested 8-chloroquinobenzothiazines **1**–**17** are characterized by poor solubility in water, which is due to their chemical structure (four six-membered condensed rings). Parameter values range from −6.26 (compound **12**) to −4.92 (compound **3**). The value of this parameter for chlorpromazine **18** used as a reference is −4.89 (Table 6). Due to the functional and morphological similarity of Caco-2 cells to the human intestinal epithelium, the study of the permeability of compounds through the monolayer of Caco-2 cells is the most commonly used in vitro method to determine absorption of orally administered drugs [44,45].

The Caco-2 permeability calculated using the pkCSM algorithms is given as the logarithm of the apparent permeability coefficient (log Papp). Compounds for which the calculated log Papp at 10^−6^ cm/s is greater than 0.9 are considered to have high Caco-2 permeability. Based on the results obtained for all tested 8-chloroquinobenzothiazines **1**–**17** (Table 6), it can be predicted that they will show high Caco-2 cell permeability (values ranged from 0.97 to 1.18). The calculated intestinal absorption values are given in percentages and indicate what percentage of the substance will be absorbed in the human intestine. The values obtained for the tested substances **1**–**17** showed that all the compounds have a very high probability of high intestinal absorption; for all the compounds the value of this parameter was greater than 90 and close to the value obtained for chlorpromazine **18**. Also, skin permeability is considered as an essential parameter to be considered when delivering active substances. It is also a parameter that makes it possible to determine the risk of using a given substance [46,47].

This parameter obtained from the pkCSM program is expressed as logKp and a compound is considered to have relatively low skin permeability if its logKp > −2.5. For all the tested substances, the obtained value of this parameter is in the range of −2.74 to −2.61 and indicates poor permeability (Table 6).

A key parameter influencing the bioavailability of a biologically active substance is its solubility in water. Substances that are poorly soluble in body fluids are difficult to dissolve, which reduces their bioavailability. In this case, various strategies can be used to increase solubility and improve absorption (formulation modifications, the use of specific carriers) [48].

The obtained values of the solubility parameter for all the new 17 compounds indicate their poor solubility, which results from their chemical structure. The obtained values of the parameters range from −6.30 for quinobenzothiazine **15** to −5.12 for quinobenzothiazine **4**. For chlorpromazine, this parameter was −4.89 (Table 6). The absorption parameters of the tested quinobenzothiazines were also evaluated for potential interactions with *p*-glycoproteins. Almost all of them (except substances **8** and **10**) can be substrates for *p*-glycoprotein, and all of them can be inhibitors of *p*-glycoprotein I and *p*-glycoprotein II (Table S3).

Using the pkCSM program, the unbound fraction (Fu) parameter was also calculated (Table 7). It is also a pharmacokinetic parameter that affects the effectiveness of the drug and any side effects that may occur (glomerular filtration in the kidneys, total clearance hepatic metabolism). Determining this parameter is of great importance because the part of the drug that has not been bound to the molecular target may lead to interactions with other proteins, enzymes, and receptors [49].

For the tested 6-substituted 8-chloroquinobenzothiazines **1**–**17**, the value of this parameter is in the range of 0.02 for compound **9** to 0.16 for compound **17**. For chlorpromazine used in the study as a reference substance, a value of 0.08 was obtained. The results indicate a low content of the unbound fraction in plasma. The obtained results indicate that the largest amount of unbound substance in plasma may remain in the case of compounds **11** and **17** containing *p*-toluenesulfonamide substituents.

For medicinal substances, it is also important to determine the degree to which they cross the blood–brain barrier. This parameter is given as logBB and is defined as the logarithmic ratio of the drug concentration in the brain to its concentration in the plasma. In the pkCSM calculation model, a substance can cross the blood–brain barrier if logBB is greater 0.3. If the obtained value of this parameter is less than −1, the substance is distributed to the brain to a small extent [50,51]. The calculated permeability through the blood–brain barrier for the tested substances **1**–**17** ranges from −0.36 (for compound **16**) to 0.58 (for compound **1**). Values greater than 0.3 were obtained for substances **1**–**5** having dialkylaminoalkyl substituents at the thiazine nitrogen atom, similarly to chlorpromazine **18** used as a reference (logBB = 0.89). According to the predictions obtained, the remaining substances will have poor permeability of the blood–brain barrier.

Permeability to the central nervous system is given as log PS. The value of this parameter obtained using the pkCSM program ranges from −2.16 for derivative **16** to −1.42 for derivative **1**, so 8-chloroquinobenzothiazines **1**–**9** and **11**–**14** can penetrate the central nervous system. Substances with log PS > −2 are considered to penetrate the central nervous system, while substances with log PS < −3 do not (Table 7, Figure S1).

An important parameter influencing the metabolism of xenobiotics (phase I of metabolism) is their susceptibility to catalysis by cytochrome P450 (CYP) enzymes. If a substance is a substrate for this enzyme, it is converted into metabolites by binding to the active site of the enzyme; however, inhibitors may be their substrates or non-substrates. It is believed that the CYP3A, CYP1A2, CYP2C9, CYP2C19, and CYP2D6 isoforms of this enzyme, occurring primarily in the liver and intestinal wall, have a large share in drug metabolism. Predictions of the interactions of the tested quinobenzothiazines with these enzymes obtained from the pkCSM program gave varied results. All quinobenzothiazines can be CYP3A4 substrates, while only three (compounds **1**, **3**, and **4**) are substrates for the CYP2D6 isoform). However, all the tested compounds may be CYP3A4 inhibitors and almost all (except compounds **11** and **17**) may be CYP1A2 inhibitors (Table S4). As is known, chlorpromazine is extensively metabolized in the liver and kidneys, and its metabolism is mainly due to cytochrome P450 isoenzymes: CYP2D6, CYP1A2, and CYP3A4.

One of the most important pharmacokinetic parameters to consider when designing drug candidates is the clearance rate. Clearance is divided into three general categories: metabolic conversion, renal excretion, and hepatobiliary excretion. The mechanism determining the clearance rate is determined by the physicochemical properties of the substance, with lipophilic molecules tending to be metabolized and hydrophilic, and polar molecules tending to be passively or actively excreted. This is one of the main parameters describing the elimination of a substance from the body, which means the volume of plasma from which substances are removed per unit of time. Using the pkCSM program, the total clearance (CLtot) can be calculated, which determines the efficiency of drug elimination from the entire body without indicating specific elimination mechanisms. This parameter is useful, among other things, for determining the dosing rate [52,53]. Among the 8-chloroquinobenzothiazines studied, CLtot values were diverse and ranged from 0.03 (for compound **17**) to 0.77 mL/min/kg (for compound **3**). The highest values, similar to the total clearance values for the chlorpromazine **18**, were obtained for derivatives with dialkylaminoalkyl substituents.

For the tested 8-chloroquinobenzothiazines, the maximum tolerated dose, acute oral toxicity in rats, T. Pyriformis toxicity, and toxicity to fish were also calculated. The obtained results are summarized in Table 8. The maximum recommended tolerated dose (MRTD) allows for the preliminary determination of the toxic dose of a given substance for humans. The obtained results of the MRTD parameter values are given as log (mg/kg/day). Obtaining this parameter facilitates the determination of the maximum recommended initial dose. According to the model used in the pkCSM program, if the calculated value is less than or equal to 0.48 log (mg/kg/day), it is considered low, and if it is higher, it is considered high. For most of the tested derivatives (**1**–**5**, **7**–**10**, **13**–**16**), the obtained results indicate a low maximum recommended dose. To determine in silico toxicity in the pkCSM program, a model based on T. Pyriformis, a protozoan bacterium, is used, the toxicity of which is considered a toxic endpoint. The value of this parameter is designated as plGC_50_ (negative logarithm of the concentration required to inhibit 50% of growth in log µg/L). The tested compound can be assessed as toxic if the plGC_50_ value is greater than −0.5 log µg/L. On the other hand, the toxicity parameter for roach is given as LC_50_, i.e., the concentration of the substance necessary to cause death of 50% of the flathead roach. According to this model, a substance for which the LC_50_ value is less than 0.5 mM (logLC_50_ < −0.3) is considered highly toxic. Hepatotoxicity and no skin sensitization are predicted for all the tested substances (similar to chlorpromazine **18**) (Table S5).

Comparing the log P_TLC_ data obtained with the results of cytotoxicity tests against various cell lines (A549, HCT-116, MiaPaCa-2), it was observed that compounds with moderate lipophilicity (log P_TLC_ in the range of 4.6–5.2) showed the highest antitumor activity while maintaining high selectivity against normal cells (HaCaT). For example, compound **2** (log P_TLC_ = 5.05) showed strong cytotoxicity against A549 (IC_50_ = 8.2 µM) and HCT-116 (IC_50_ = 1.6 µM) cells, achieving high selectivity indices of 7.6 and 39, respectively. A similar profile was shown by compound **9** (log P_TLC_ = 4.66), which achieved the highest SI for the A549 line (SI = 10.7). Particularly outstanding was compound **16** (log P_TLC_ = 4.90), which selectively and strongly inhibited the growth of HCT-116 cells (IC_50_ = 0.7 µM, SI = 143), while showing a lack of toxicity to non-cancer cells. In contrast, excessive lipophilicity, as in the case of compound **17** (log P_TLC_ = 6.22), although correlated with good activity against A549 (IC_50_ = 9.45 µM), no longer led to equally favorable SI values against other lines. Compound **15** (log P_TLC_ = 4.82), despite its low IC_50_ value (6.98 µM), had very low selectivity (SI = 0.1), indicating its potential non-selective toxicity.

## 3. Materials and Methods

### 3.1. Analytes

The chemical structures of the investigated compounds are shown in Figure 1. The details of their synthesis including the results of spectroscopic studies by using ^1^H NMR, ^13^C NMR (Bruker, Billerica, MA, USA), and HRMS (Bruker, Billerica, MA, USA). techniques have been described in previous work [32].

### 3.2. Reagents and Materials

Ethanol (96%, Reag. Ph Eur.) used to dissolve analytes was purchased from POCh (Gliwice, Poland). The mobile phase component acetone (HPLC grade) was obtained from POCh (Gliwice, Poland) and buffer TRIS (tris(hydroxymethyl)aminomethane) at pH = 7.4 was obtained from Fluka (Buchs, Switzerland). All chromatographic analyses were carried out using the TLC method on aluminum plates (20 cm × 20 cm) precoated with silica gel 60 RP-18F_254_ manufactured by Merck (Darmstadt, Germany).

### 3.3. Determination of Lipophilicity Descriptors Using the TLC Method

The chromatographic parameter of lipophilicity was determined by means of Soczewiński–Wachtmeister’s method [9]. A mixture of acetone and TRIS buffer (pH = 7.4) was used as the mobile phase to determine the lipophilicity chromatographic parameters (R_M0_) of the tested compounds **1**–**18** under physiological conditions. The acetone ratio was 50 to 85% (*v*/*v*) in increments of 5%. The tested compounds in the form of an ethanol solution at a concentration of 2.0 mg/mL each were spotted on chromatographic plates at an amount of 2 µL each. Before analysis, the chromatography chamber (Camag, Switzerland) was saturated with the mobile phase (50 mL) for 30 min. After development at 20 ± 2 °C and subsequent drying, the chromatograms were observed under UV light at 254 nm by using a Camag UV lamp (Muttenz, Switzerland). Each analysis was performed in triplicate. The average value of the retardation factor (Rf) was used to calculate R_M_ using Equation (1):

(1) $$R_{M}=log(\frac{1-R_{f}}{R_{f}})$$

Next, the linear relationships between the obtained R_M_ values and the concentration of acetone in the mobile phase used allowed the determination of the chromatographic parameters of lipophilicity for investigated compounds **1**–**18**:

(2) $$R_{M}=\;R_{M0}+b\times C$$

where C—acetone concentration in the mobile phase; b—the slope of linear regression plot.

In addition, the chromatographic parameter of lipophilicity R_M0_ and b value allowed the determination of the chromatography hydrophobic index C_0_ by using the following formula:

(3) $$C_{0}=-\;\frac{R_{M0}}{b}$$

### 3.4. In Silico Calculations

In our study, the SwissADME web tool freely accessible at http://www.swissadme.ch (accessed on 2 January 2025) as well as computer program ChemDraw was used for calculations of log P values (iLOGP, XLOGP3, WLOGP, MLOGP, SILCOS-IT, and Clog P) for all the studied compounds [27,33,34]. The information regarding algorithms and suppliers is presented in the Supplementary Materials (Table S6).

The in silico calculation programs used perform calculations based on SMILES (Simplified Molecular Input System) formulas; therefore, before starting the calculations, the structural formulas of the studied substances were converted into such formulas using the ChemDraw program (Perkin Elmer Informatics, Waltham, MA, USA). The formulas are presented in Table S7.

In addition, the molecular descriptors and other ADME parameters of these 18 compounds were also obtained using the SwissADME (MW, n-HA, n-ArHA, n-ROT, n-HBA, n-HBD, MR, TPSA, LogKp) and pkCSM platforms (water solubility, Caco2 permeability, intestinal absorption, skin permeability, VDss, fraction unbound, BBB permeability, CNS permeability, total clearance, max. tolerated dose, oral rat acute toxicity, oral rat chronic toxicity, *t*. pyriformis toxicity, minnow toxicity) [42]. All the calculated log P values are presented in Table 1.

### 3.5. Statistical Evaluation of the Data

The correlation matrix of the obtained lipophilicity parameters as well as cluster analysis was performed using Statistica program version 13.3. The results are presented in the form of linear equation correlations and dendrograms (based on Euclidean distance) [54].

## 4. Conclusions

The aim of the work was to estimate the selected parameters including lipophilicity of seventeen newly synthesized quinoline derivatives of chlorpromazine, which are crucial for describing the ADME profile of these drug candidates. The lipophilicity parameters of the tested compounds were determined by using an experimental method, i.e., the RP-TLC technique and using different computational tools. Analysis of the experimentally obtained lipophilicity parameters expressed as log P_TLC_ indicates that the highest lipophilicity was determined for the derivative with a *p*-toluenesulfonamidobutyl substituent at the thiazine nitrogen atom and the lowest was for the derivative with an acet-ylaminopropyl substituent. In addition, compounds with the butylene chain were more lipophilic than compounds with the propylene linker. Among the tested compounds, those with moderate experimental lipophilicity (log P_TLC_ ~4.6–5.2) exhibited the most favorable profiles, achieving potent anticancer activity with high selectivity indices (SI), particularly against HCT-116 and A549 cancer cell lines. Notably, compound **16** (log P_TLC_ = 4.90) demonstrated exceptional selectivity (SI = 143) and cytotoxicity (IC_50_ = 0.7 µM), while sparing non-cancerous cells. In contrast, compounds with excessively high lipophilicity (e.g., compound **17**, log P_TLC_ = 6.22), though still cytotoxic, displayed reduced selectivity, likely due to non-specific interactions with cell membranes. These results may indicate a measurable relationship between lipophilicity and selective cytotoxic activity, suggesting the existence of an optimal lipophilicity window for balancing cell membrane permeability and off-target toxicity. This knowledge provides a valuable framework for the design of future phenothiazine-based anticancer drugs, enabling the initial selection of candidate molecules based on predicted or experimentally determined lipophilicity parameters.

The strong linear relationship between the experimental parameter of lipophilicity (log P_TLC_) and calculated lipophilicity factor expressed as Clog P for the studied compounds shows that the linear equation determined between the two variables can be useful in rapid prediction of the experimental parameter of lipophilicity of studied compounds without conducting experiments. Cluster analysis of the lipophilicity parameters of the tested compounds and the computational programs used to predict theoretical log P values confirmed similarities between certain compounds as well as among specific calculation methods. The greatest similarity among all the calculated log P values is indicated by the partition coefficient expressed as SILICOS-IT and MLOGP. They form one cluster with a small Euclidean distance. The chromatographic parameter of lipophilicity of the tested compounds (log P_TLC_) shows the similarity to them and to the XLOGP3 values of the tested substances.

Our work also confirms the usefulness of in silico methods as an inexpensive and rapid predictive tool for the preliminary assessment of the ADME profile including the factors responsible for absorption (e.g., water solubility, Caco-2 permeability, intestinal absorption, skin permeability), distribution (VDss, unbound fraction, BBB permeability, and OUN permeability), excretion, and toxicity (total clearance max. tolerated dose, acute food toxicity for rats, chronic food toxicity for rats, T. Pyriformis toxicity and minnow toxicity) for the discussed substances. The conducted study of lipophilicity and ADME factors allowed us to confirm that the studied compounds obtained in multi-stage reactions based on 6-*H*-8-chloroquinobenzothiazine exhibit beneficial properties of ADME and can be considered as promising starting structures for further studies. The results of the lipophilicity–cytotoxicity analysis suggest that moderate lipophilicity represents a favorable compromise between permeability across biological membranes and cytotoxic selectivity. Furthermore, they confirm that lipophilicity parameters, including experimental log P_TLC_, are valuable criteria in the design of new phenothiazine derivatives with targeted anticancer activity. In the next stages of the study, experimental studies will be conducted, which are necessary to confirm these ADMET parameters and the biological activity of the most promising structures developed in our laboratory.

## Figures and Tables

**Table 1:** The computed lipophilicity parameters (log P_calc_) for 8-chloroquinobenzothiazines **1**–**17** and chlorpromazine **18** using the internet data bases: SwissADME [33] and ChemDraw (Version 22.2.0) [34].

No. of Compound	iLOGP	XLOGP3	WLOGP	MLOGP	SILICOS-IT	Clog P
**1**	4.18	5.75	5.45	4.85	4.83	7.04
**2**	3.94	5.33	5.06	4.63	4.44	6.29
**3**	4.11	5.50	4.83	4.85	4.71	6.55
**4**	4.16	5.86	5.22	5.06	4.94	7.11
**5**	4.23	6.29	5.60	5.27	5.04	7.49
**6**	3.94	6.01	5.42	4.66	4.66	7.52
**7**	3.06	4.65	4.64	4.11	4.42	5.25
**8**	3.46	5.07	5.04	4.53	4.55	6.56
**9**	3.50	5.42	5.25	4.31	4.36	6.29
**10**	2.53	4.44	3.15	3.39	3.33	5.43
**11**	3.69	6.36	6.87	4.73	4.90	7.60
**12**	4.18	6.37	5.81	5.11	5.69	7.90
**13**	3.64	5.01	5.03	4.01	4.81	5.31
**14**	4.29	5.78	5.64	3.04	4.57	6.76
**15**	3.59	5.43	5.43	4.05	4.95	6.43
**16**	3.43	4.80	5.52	3.34	3.72	5.52
**17**	3.67	6.72	7.26	4.65	5.30	7.81
**18**	3.47	5.19	4.51	4.35	3.94	5.80

**Table 2:** R_M0_ and log P_lit_ values and b (slope) and r (correlation coefficient) of the equation R_M_ = R_M0_ + bC for standards.

Reference Compound	Lipophilicity Parameters				
	log P_lit_	R_M0_	−b	r	log P_TLC_
acetanilide	1.21 [35]	0.78	0.0162	0.9923	1.21
benzoic acid	1.87 [36]	1.16	0.0247	0.9937	1.70
benzophenone	3.18 [36]	2.51	0.0328	0.9971	3.43
anthracene	4.45 [36]	3.33	0.0412	0.9982	4.49
p,p’-DDT	6.38 [37]	4.69	0.0564	0.9977	6.24

**Table 3:** The experimental lipophilicity parameters (log P_TLC_ values) for compounds **1**–**18**.

No. of Compound	log P_TLC_	No. of Compound	log P_TLC_	No. of Compound	log P_TLC_
**1**	5.33	**7**	3.70	**13**	4.21
**2**	5.05	**8**	5.04	**14**	5.43
**3**	5.29	**9**	4.66	**15**	4.82
**4**	5.36	**10**	4.53	**16**	4.90
**5**	5.61	**11**	6.10	**17**	6.22
**6**	5.50	**12**	5.88	**18**	4.59

**Table 4:** Drug-likeness and ADME properties predicted by in silico studies using SwissADME.

No.	MW (g/mol)	n-HA	n-ArHA	n-ROT	n-HBA	n-HBD	MR	TPSA [Å^2^]	LogKp [cm/s]
**1**	383.94	26	16	5	2	0	115.19	44.67	−4.56
**2**	369.91	25	16	4	2	0	110.38	44.67	−4.74
**3**	381.92	26	16	3	2	0	116.99	108.64	−4.72
**4**	395.95	27	16	3	2	0	121.79	44.67	−4.55
**5**	409.97	28	16	3	2	0	126.60	44.67	−4.33
**6**	471.96	33	22	4	3	0	137.67	78.81	−4.91
**7**	383.89	26	16	5	2	1	110.49	70.53	−5.34
**8**	413.92	28	16	7	3	1	116.77	76.76	−5.34
**9**	447.38	29	16	8	2	2	123.28	82.56	−5.43
**10**	419.95	27	16	5	4	1	114.44	95.98	−5.71
**11**	496.06	33	22	6	4	1	138.25	95.98	−4.81
**12**	485.98	34	22	5	3	0	142.48	78.81	−4.74
**13**	397.92	27	16	6	2	1	115.29	70.53	−5.17
**14**	427.95	29	16	8	3	1	121.57	79.76	−4.81
**15**	461.41	30	16	9	2	2	128.09	82.56	−5.26
**16**	433.97	28	16	6	4	1	119.25	95.98	−5.54
**17**	510.07	34	22	7	4	1	143.06	95.98	−4.64
**18**	318.86	21	12	4	1	0	95.05	31.78	−4.56

MW: molecular weight; n-HA: number of heavy atoms; n-ArHA: number of arom. heavy atoms; n-ROT: number of rotatable bonds; n-HBA: number of hydrogen bond acceptors; n-HBD: number of hydrogen bond donors; MR: molar refractivity; TPSA: topological polar surface area; LogKp: skin permeability.

**Table 5:** Properties of examined compounds based on analysis of Lipinski’s, Ghose’s, Veber’s, Egan’s, and Muegge’s rules.

No.	Lipinski’s Rules	Ghose’s Rules	Veber’s Rules	Egan’s Rules	Muegge’s Rules
**1**	+	+	+	+	- (XLOGP3 > 5)
**2**	+	+	+	+	- (XLOGP3 > 5)
**3**	+	+	+	+	- (XLOGP3 > 5)
**4**	+	+	+	+	- (XLOGP3 > 5)
**5**	+	- (WLOGP > 5.6)	+	+	- (XLOGP3 > 5)
**6**	+	- (MR > 130)	+	+	- (XLOGP3 > 5)
**7**	+	+	+	+	+
**8**	+	+	+	+	- (XLOGP3 > 5)
**9**	+	+	+	+	- (XLOGP3 > 5)
**10**	+	+	+	+	+
**11**	+	- (MW > 480. WLOGP > 5.6. MR > 130)	+	- (WLOGP > 5.88)	- (XLOGP3 > 5)
**12**	+	- (MW > 480. WLOGP > 5.6. MR > 130)	+	+	- (XLOGP3 > 5)
**13**	+	+	+	+	- (XLOGP3 > 5)
**14**	+	- (WLOGP > 5.6)	+	+	- (XLOGP3 > 5)
**15**	+	+	+	+	- (XLOGP3 > 5)
**16**	+	+	+	+	+
**17**	- (MW > 500. MLOGP > 4.15)	- (MW > 480. WLOGP > 5.6. MR > 130)	+	- (WLOGP > 5.88)	- (XLOGP3 > 5)
**18**	+	+	+	+	- (XLOGP3 > 5)

Lipinski’s Rule: MW ≤ 500; MLOGP ≤ 4.15; n-HBA ≤ 10; n-HBD ≤ 5. Ghose’s Rule (160 ≤ MW ≤ 480; 40 ≤ MR ≤ 130; −0.4 ≤ WLOGP ≤ 5.6; 20 ≤ Atoms ≤ 70. Veber’s Rule: n-ROT ≤ 10; TPSA ≤ 140 Å^2^. Egan’s Rules: WLOG ≤ 5.88. TPSA ≤ 131.6 Å^2^. Muegge’s Rules: MW ≤ 600; −2 ≤ XLOGP3 ≤ 5; TPSA ≤ 150 Å^2^; num. rings ≤ 7; num. carbons > 4; num. heteroatoms ≥ 1; n-ROT ≤ 15; nHBA ≤ 10; nHBD ≤ 5.

**Table 6:** The absorption descriptors for 6-substituted 8-chloroquinobenzothiazines **1**-**17** and chlorpromazine **18**.

No. of Compound	Water Solubility [log mol/L]	Caco2 Permeability [log Papp in 10^−6^ cm/s]	Intestinal Absorption [% Absorbed]	Skin Permeability [log Kp]
**1**	−5.31	0.97	91.62	−2.63
**2**	−5.13	1.03	93.86	−2.61
**3**	−4.92	0.98	92.32	−2.66
**4**	−5.12	0.98	91.93	−2.66
**5**	−5.36	1.02	93.37	−2.65
**6**	−5.94	1.06	93.65	−2.73
**7**	−5.41	1.06	94.54	−2.63
**8**	−5.88	1.02	93.17	−2.67
**9**	−5.94	1.01	91.90	−2.73
**10**	−5.28	1.18	94.53	−2.69
**11**	−5.72	1.11	93.43	−2.74
**12**	−6.22	1.05	93.53	−2.73
**13**	−5.80	1.05	94.56	−2.62
**14**	−6.23	1.02	93.09	−2.66
**15**	−6.30	1.00	91.93	−2.72
**16**	−5.64	1.14	94.45	−2.67
**17**	−5.92	1.07	93.36	−2.73
**18**	−4.89	1.48	93.52	−2.57

**Table 7:** The distribution descriptors for 6-substituted 8-chloroquinobenzothiazines **1**–**17** and chlorpromazine **18**.

No. of Compound	VDss [log L/kg]	Fraction Unbound [Fu]	BBB Permeability [log BB]	CNS Permeability [log PS]
**1**	1.50	0.10	0.58	−1.42
**2**	1.51	0.10	0.509	−1.53
**3**	1.46	0.12	0.49	−1.43
**4**	1.51	0.11	0.477	−1.42
**5**	1.67	0.10	0.429	−1.55
**6**	0.22	0.10	0.029	−1.48
**7**	0.53	0.06	−0.07	−1.59
**8**	0.48	0.04	−0.035	−1.83
**9**	0.40	0.02	−0.15	−1.97
**10**	0.31	0.05	−0.27	−2.15
**11**	0.12	0.15	−0.12	−1.72
**12**	0.24	0.11	−0.05	−1.52
**13**	0.63	0.05	−0.13	−1.66
**14**	0.58	0.04	−0.22	−1.84
**15**	0.49	0.01	−0.32	−2.03
**16**	0.42	0.04	−0.34	−2.16
**17**	0.12	0.16	−0.30	−1.74
**18**	1.83	0.08	0.89	−1.38

**Table 8:** The excretion and toxicity for 6-substituted 8-chloroquinobenzothiazines **1**–**17** and chlorpromazine **18**.

No. of Compound	Total Clearance [log ml/min/kg]	Max. Tolerated Dose [log g/kg/day]	Oral Rat Acute Toxicity [mol/kg]	Oral Rat Chronic Toxicity [log mg/kg bw/day]	*T. Pyriformis* Toxicity [log μg/L]	Minnow Toxicity [log mM]
**1**	0.72	0.38	2.30	0.92	0.92	−0.60
**2**	0.60	0.34	2.96	1.11	1.11	−0.58
**3**	0.77	0.13	3.07	0.98	0.47	−0.14
**4**	0.72	0.14	3.10	0.90	0.45	−0.25
**5**	0.68	0.18	3.13	0.91	0.43	−0.52
**6**	0.27	0.56	2.81	0.68	0.29	−5.09
**7**	0.19	0.22	2.71	0.87	0.57	−1.76
**8**	0.29	0.32	2.60	0.82	0.49	−2.75
**9**	0.26	0.37	2.37	1.31	0.35	0.64
**10**	0.29	0.34	2.54	2.54	0.47	−3.13
**11**	0.07	0.69	2.78	0.57	0.29	−4.81
**12**	0.24	0.52	2.75	0.69	0.29	−4.20
**13**	0.16	0.28	2.76	1.20	0.52	−0.98
**14**	0.26	0.38	2.68	0.79	0.46	−1.93
**15**	0.23	0.42	2.38	1.48	0.34	1.78
**16**	0.26	0.39	2.63	0.76	0.44	−2.35
**17**	0.03	0.69	2.75	0.53	0.29	−3.71
**18**	0.67	0.36	3.02	0.77	2.16	−0.94

## Data Availability

The original contributions presented in this study are included in the article/Supplementary Materials. Further inquiries can be directed to the corresponding author.

## Supplementary Materials

The following supporting information can be downloaded at: https://www.mdpi.com/article/10.3390/biom15081194/s1, Table S1: Cytotoxic activity (IC_50_, μM) of studied compounds estimated by the MTT assay; Table S2: Data for linear correlation (R_M_ = R_M0_ + bC) for compounds **1**–**18**; Table S3: The absorption descriptors for 6-substituted 8-chloroquinobenzothiazines **1**–**17** and chlorpromazine **18** [31]; Table S4: The metabolism descriptors for 6-substituted 8-chloroquinobenzo-thiazines **1**–**17** and chlorpromazine **18**; Table S5: The excretion and toxicity for 6-substituted 8-chloroquinobenzo-thiazines **1**–**17** and chlorpromazine **18**; Table S6: List of software used to determine theoretical logP values for tested compounds; Table S7: Structural formulas and SMILES formulas of the tested substances **1**–**18**; Figure S1: Boiled-Egg representation of the intestinal absorption and the permeation through blood-brain barrier for diquinothiazines **1**–**17** and chlorpromazine **18**.
