# Supplementary material for: Study of the Lipophilicity of Tetracyclic Anticancer Azaphenothiazines
Source: Biomolecules. 2025 Aug 19;15(8):1194. doi: 10.3390/biom15081194 (PMC12384454; doi:10.3390/biom15081194)
Supplement: Supplementary file 1 [file biomolecules-15-01194-s001.zip › biomolecules-3740796-supplementary.pdf]

Study of the Lipophilicity of Tetracyclic Anticancer Azaphenothiazines

Małgorzata Jeleń [1]*, Beata Morak-Młodawska [1], Małgorzata Dołowy [2] and Adam Konefał [3]

[1] Department of Organic Chemistry, Faculty of Pharmaceutical Sciences in Sosnowiec, Medical University of Silesia in Katowice, Jagiellońska Street 4, 41-200 Sosnowiec, Poland; manowak@sum.edu.pl (M.J.); bmlodawska@sum.edu.pl. (B.M.M.)
[2] Department of Analytical Chemistry, Faculty of Pharmaceutical Sciences in Sosnowiec, Medical University of Silesia in Katowice, Jagiellońska 4, 41-200 Sosnowiec, Poland; mdolowy@sum.edu.pl (M.D.)
[3] Institute of Physics, University of Silesia in Katowice, 40-007 Katowice, Poland; adam.konefal@us.edu.pl (A.K.)
* Correspondence: manowak@sum.edu.pl (MJ)

Table S1. Cytotoxic activity (IC_{50}, μM) of studied compounds estimated by the MTT assay [31].

Compound	Cancer cells				Normal cells
	**A549**		**MDA**		**HaCaT**
	IC_{50}	SI	IC_{50}	SI	IC_{50}
**1**	27.2 ± 8.4	3.7	< 100	1.0	< 100
**2**	8.2 ± 3.6	7.6	52.1 ± 2.7	1.2	62.3 ± 2.5
**3**	17.4 ± 4.1	5.7	77.1 ± 1.6	1.3	< 100
**4**	63.8 ± 8.5	1.6	< 100	1.0	< 100
**5**	16.0 ± 4.2	1	16.7 ± 1.1	0.9	12.7 ± 1.9
**7**	24.6 ± 6.1	4.1	76.6 ± 8.9	1.3	< 100
**8**	86.6 ± 0.8	1.1	< 100	1.0	< 100
**9**	9.3 ± 1.2	10.7	48.6 ± 7.3	2.0	< 100
**10**	< 100	1.0	< 100	1.0	< 100
**11**	<100	1.0	< 100	1.0	< 100
**13**	82.3 ± 6.2	1.2	< 100	1.0	< 100
**14**	<100	1.0	< 100	1.0	< 100
**15**	6.98 ± 1.2	0.1	7.4 ± 1.2	0.1	1.1 ± 0.3
**16**	30.5 ± 8.3	3.3	< 100	0.2	< 100
**17**	9.45 ± 1.3	10.5	< 100	1.0	< 100
**DX**	0.6 ± 0.2	0.14	0.8 ± 0.1	0.15	0.3 ± 0.1

Compound	Cancer cells	Normal cells

	MiaPaCa-2		PC3		HCT116		HaCaT
	IC_{50}	SI	IC_{50}	SI	IC_{50}	SI	IC_{50}
**2**	40.2 ± 0.7	1.6	52.1 ± 7.1	1.2	1.6 ± 0.8	39	62.3 ± 3.5
**3**	57.4 ± 9.6	1.7	77.1 ± 9.4	1.3	17.5 ± 1.4	5.7	< 100
**5**	24.3 ± 3.5	0.5	16.7 ± 1.8	0.7	7.7 ± 1.2	1.6	12.7 ± 2.1
**9**	23.2 ± 2.7	4.3	34.8 ± 9.8	2.8	10.4 ± 1.6	8.8	< 100
**15**	6.4 ± 2.4	0.2	76.6 ± 9.8	0.1	11.3 ± 2.2	0.1	1.1 ± 0.2
**16**	98.4 ± 5.6	1.0	< 100	1.0	0.7 ± 0.08	143	< 100
**17**	< 100	1.0	48.6 ± 4.7	2.0	< 100	1.0	< 100
**DX**	0.6 ± 0.2	0.14	0.8 ± 0.1	0.15	0.59 ± 0.02	0.5	0.3 ± 0.1

Table S2. Data for linear correlation ($R_M = R_{M0} + bC$) for compounds **1-18**

No. of compound	R_{M0}	b	r	C_0
**1**	3.98	-0.06	0.9954	69.58
**2**	3.77	-0.05	0.9956	70.60
**3**	3.96	-0.05	0.9968	73.88
**4**	4.01	-0.05	0.9964	73.04
**5**	4.20	-0.06	0.9973	84.51
**6**	4.12	-0.05	0.9952	82.90
**7**	2.72	-0.04	0.9982	76.19
**8**	3.76	-0.05	0.9973	81.74
**9**	3.47	-0.05	0.9968	76.43
**10**	3.37	-0.04	0.9961	76.24
**11**	4.59	-0.06	0.9952	79.96
**12**	4.41	-0.05	0.9980	85.47
**13**	3.11	-0.04	0.9927	77.17
**14**	4.06	-0.05	0.9972	82.86
**15**	3.59	-0.05	0.9954	76.22
**16**	3.65	-0.05	0.9936	76.36
**17**	4.68	-0.06	0.9977	80.41
**18**	3.41	-0.05	0.9912	67.79

Table S3. The absorption descriptors for 6-substituted 8-chloroquinobenzothiazines **1–17** and chlorpromazine **18**.

No. of compound	P-glycoprotein substrate	P-glycoprotein I inhibitor	P-glycoprotein II inhibitor
**1**	+	+	+
**2**	+	+	+
**3**	+	+	+
**4**	+	+	+
**5**	+	+	+
**6**	+	+	+
**7**	+	+	+
**8**	-	+	+
**9**	+	+	+
**10**	-	+	+
**11**	+	+	+
**12**	+	+	+
**13**	+	+	+
**14**	+	+	+
**15**	+	+	+
**16**	+	+	+
**17**	+	+	+
**18**	+	+	+

Table S4. The metabolism descriptors for 6-substituted 8-chloroquinobenzothiazines **1–17** and chlorpromazine **18**.

No. of compound	CYP2D6 substrate	CYP3A4 substrate	CYP1A2 inhibitior	CYP2C19 inhibitior	CYP2C9 inhibitior	CYP2D6 inhibitior	CYP3A4 inhibitior
**1**	+	+	+	+	-	-	+
**2**	-	+	+	-	-	+	+
**3**	+	+	+	-	-	+	+
**4**	+	+	+	-	-	+	+
**5**	-	+	+	-	-	+	+
**6**	-	+	+	+	+	-	+
**7**	-	+	+	+	+	-	+
**8**	-	+	+	+	+	-	+
**9**	-	+	+	+	+	-	+
**10**	-	+	+	+	+	-	+
**11**	-	+	-	+	+	-	+
**12**	-	+	+	+	+	-	+
**13**	-	+	+	+	+	-	+
**14**	-	+	+	+	+	-	+
**15**	-	+	+	+	+	-	+
**16**	-	+	+	+	+	-	+
**17**	-	+	-	+	+	-	+
**18**	+	+	+	-	-	+	-

Table S5. The excretion and toxicity for 6-substituted 8-chloroquinobenzothiazines **1–17** and chlorpromazine **18**.

No. of compound	Renal OCT2 substrate	AMES toxicity	hERG I inhibitor	hERG II inhibitor	*Hepatotoxicity*	Skin Sensitisation
**1**	+	+	+	+	+	-
**2**	+	+	+	+	+	-
**3**	+	+	+	+	+	-
**4**	+	+	+	+	+	-
**5**	+	-	+	+	+	-
**6**	-	+	-	+	+	-
**7**	-	-	-	+	+	-
**8**	-	-	-	+	+	-
**9**	-	+	-	+	+	-
**10**	-	-	-	+	+	-
**11**	-	-	-	+	+	-
**12**	-	-	-	+	+	-
**13**	-	-	-	+	+	-
**14**	-	-	-	+	+	-
**15**	-	+	-	+	+	-
**16**	-	-	-	+	+	-
**17**	-	-	-	+	+	-
**18**	+	-	-	+	+	-

Table S6. List of software used to determine theoretical logP values for tested compounds.

logP	Algorithms	Supplier
**ClogP**	fragment contribution	ChemDraw
**SILICOS-IT**	an hybride method relying on fragments and topological descriptors	http://www.swissadme.ch
**iLOGP**	physics based method relying on free energies of solvation in n-octanol-water, calculated on two GB/SA parameters solely	http://www.swissadme.ch
**XLOGP3**	atom-base method including corrective factors	http://www.swissadme.ch
**WLOGP**	includes the purely atomistic method based on the fragmental system of Wildman and Crippen	http://www.swissadme.ch
**MLOGP**	property dependent method based on topological descriptors	http://www.swissadme.ch

Table S7. Structural formulas and SMILES formulas of the tested substances **1**-**18**.

No. of comp.	Structure	SMILES
**1**	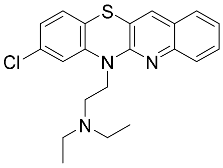	ClC1=CC(N(CCN(CC)CC)C(N=C(C=CC=C2)C2=C3)=C3S4)=C4C=C1
**2**	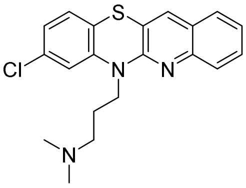	ClC1=CC(N(CCCN(C)C)C(N=C(C=CC=C2)C2=C3)=C3S4)=C4C=C1
**3**	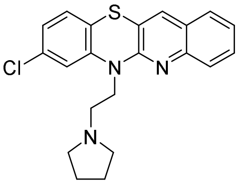	ClC1=CC(N(CCN2CCCC2)C(N=C(C=CC=C3)C3=C4)=C4S5)=C5C=C1
**4**	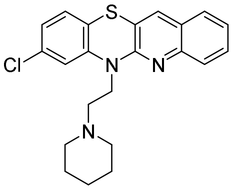	ClC1=CC(N(CCN2CCCCC2)C(N=C(C=CC=C3)C3=C4)=C4S5)=C5C=C1
**5**	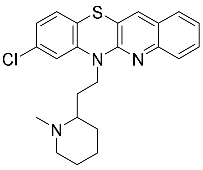	ClC1=CC(N(CCC2N(C)CCCC2)C(N=C(C=CC=C3)C3=C4)=C4S5)=C5C=C1
**6**	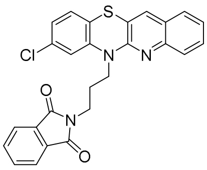	ClC1=CC(N(CCCN2C(C(C=CC=C3)=C3C2=O)=O)C(N=C(C=CC=C4)C4=C5)=C5S6)=<br>C6C=C1
**7**	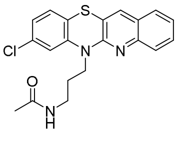	ClC1=CC(N(CCCNC(C)=O)C(N=C(C=CC=C2)C2=C3)=C3S4)=C4C=C1
**8**	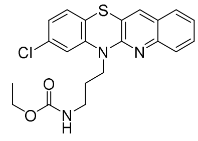	ClC1=CC(N(CCCNC(OCC)=O)C(N=C(C=CC=C2)C2=C3)=C3S4)=C4C=C1
**9**	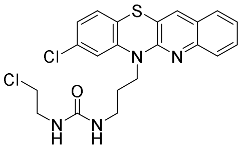	ClC1=CC(N(CCCNC(NCCCl)=O)C(N=C(C=CC=C2)C2=C3)=C3S4)=C4C=C1

**10**	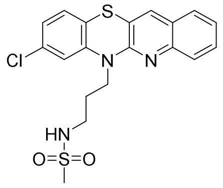	ClC1=CC2=C(C=C1)SC3=C(N=C4C=CC=CC4=C3)N2CCCNS(C)(=O)=O
**11**	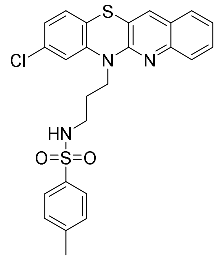	ClC1=CC2=C(C=C1)SC3=C(N=C4C=CC=CC4=C3)N2CCCNS(C5=CC=C(C)C=C5)(=O)=O
**12**	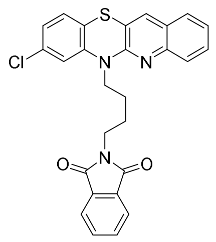	ClC1=CC(N(CCCCN2C(C(C(C=CC=C3)=C3C2=O)=O)C(N=C(C=CC=C4)C4=C5)=C5S6)=C6C=C1
**13**	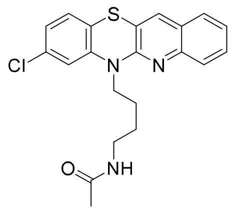	ClC1=CC(N(CCCCNC(C)=O)C(N=C(C=CC=C2)C2=C3)=C3S4)=C4C=C1
**14**	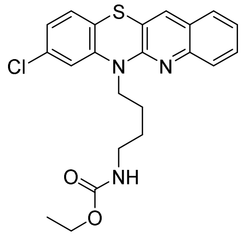	ClC1=CC(N(CCCCNC(OCC)=O)C(N=C(C=CC=C2)C2=C3)=C3S4)=C4C=C1
**15**	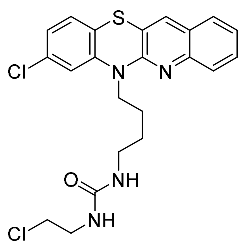	ClC1=CC(N(CCCCNC(NCCCl)=O)C(N=C(C=CC=C2)C2=C3)=C3S4)=C4C=C1
**16**	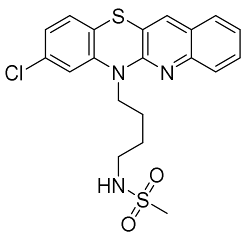	ClC1=CC2=C(C=C1)SC3=C(N=C4C=CC=CC4=C3)N2CCCCNS(C)(=O)=O

**17**	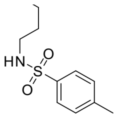	ClC1=CC2=C(C=C1)SC3=C(N=C4C=CC=CC4=C3)N2CCCCNS(C5=CC=C(C)C=C5)(=O)=O
**18**		ClC1=CC(N(CCCN(C)C)C(C=CC=C2)=C2S3)=C3C=C1

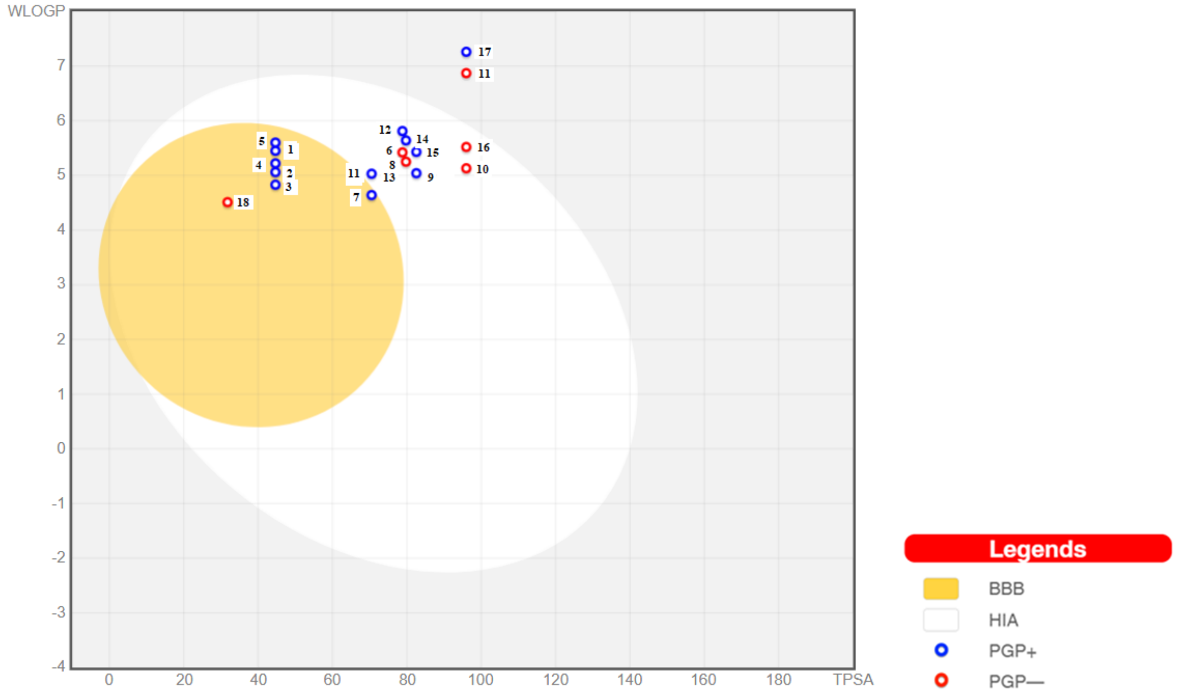

Figure S1. Boiled-Egg representation of the intestinal absorption and the permeation through blood-brain barrier for diquinothiazines **1**-**17** and chlorpromazine **18**.
